# The Association Between Engagement and Weight Loss Through Personal Coaching and Cell Phone Interventions in Young Adults: Randomized Controlled Trial

**DOI:** 10.2196/10471

**Published:** 2018-10-18

**Authors:** Pao-Hwa Lin, Steven Grambow, Stephen Intille, John A Gallis, Tony Lazenka, Hayden Bosworth, Corrine L Voils, Gary G Bennett, Bryan Batch, Jenifer Allen, Leonor Corsino, Crystal Tyson, Laura Svetkey

**Affiliations:** 1 Nephrology Division Department of Medicine Duke University Medical Center Durham, NC United States; 2 Sarah W Stedman Nutrition and Metabolism Center Duke University Medical Center Durham, NC United States; 3 Biostatistics and Bioinformatics Duke University Medical Center Durham, NC United States; 4 College of Computer and Information Science Northeastern University Boston, MA United States; 5 Bouvé College of Health Sciences Northeastern University Boston, MA United States; 6 Duke Global Health Institute Duke University Medical Center Durham, NC United States; 7 Population Health Sciences Duke University Medical Center Durham, NC United States; 8 Center for Health Services Research in Primary Care Veterans Affairs Medical Center Durham, NC United States; 9 School of Nursing Duke University Medical Center Durham, NC United States; 10 Department of Psychiatry School of Medicine Duke University Medical Center Durham, NC United States; 11 Department of Medicine School of Medicine Duke University Medical Center Durham, NC United States; 12 William S Middleton Memorial Veterans Hospital Madison, WI United States; 13 School of Medicine and Public Health University of Wisconsin Madison, WI United States; 14 Global Digital Health Science Center Duke University Medical Center Durham, NC United States; 15 Department of Psychology & Neuroscience Duke University Medical Center Durham, NC United States; 16 Division of Endocrinology, Metabolism, and Nutrition Department of Medicine Duke University Medical Center Durham, NC United States; 17 Clinical & Translational Science Institute Duke University Medical Center Kannapolis, NC United States

**Keywords:** mHealth, mobile health, weight reduction, intervention, smartphone, mobile phone

## Abstract

**Background:**

Understanding how engagement in mobile health (mHealth) weight loss interventions relates to weight change may help develop effective intervention strategies.

**Objective:**

This study aims to examine the (1) patterns of participant engagement overall and with key intervention components within each intervention arm in the Cell Phone Intervention For You (CITY) trial; (2) associations of engagement with weight change; and (3) participant characteristics related to engagement.

**Methods:**

The CITY trial tested two 24-month weight loss interventions. One was delivered with a smartphone app (cell phone) containing 24 components (weight tracking, etc) and included prompting by the app in predetermined frequency and forms. The other was delivered by a coach via monthly calls (personal coaching) supplemented with limited app components (18 overall) and without any prompting by the app. Engagement was assessed by calculating the percentage of days each app component was used and the frequency of use. Engagement was also examined across 4 weight change categories: gained (≥2%), stable (±2%), mild loss (≥2% to <5%), and greater loss (≥5%).

**Results:**

Data from 122 cell phone and 120 personal coaching participants were analyzed. Use of the app was the highest during month 1 for both arms; thereafter, use dropped substantially and continuously until the study end. During the first 6 months, the mean percentage of days that any app component was used was higher for the cell phone arm (74.2%, SD 20.1) than for the personal coaching arm (48.9%, SD 22.4). The cell phone arm used the apps an average of 5.3 times/day (SD 3.1), whereas the personal coaching participants used them 1.7 times/day (SD 1.2). Similarly, the former self-weighed more than the latter (57.1% days, SD 23.7 vs 32.9% days, SD 23.3). Furthermore, the percentage of days any app component was used, number of app uses per day, and percentage of days self-weighed all showed significant differences across the 4 weight categories for both arms. Pearson correlation showed a negative association between weight change and the percentage of days any app component was used (cell phone: *r*=−.213; personal coaching: *r*=−.319), number of apps use per day (cell phone: *r*=−.264; personal coaching: *r*=−.308), and percentage of days self-weighed (cell phone: *r*=−.297; personal coaching: *r*=−.354). None of the characteristics examined, including age, gender, race, education, income, energy expenditure, diet quality, and hypertension status, appeared to be related to engagement.

**Conclusions:**

Engagement in CITY intervention was associated with weight loss during the first 6 months. Nevertheless, engagement dropped substantially early on for most intervention components. Prompting may be helpful initially. More flexible and less intrusive prompting strategies may be needed during different stages of an intervention to increase or sustain engagement. Future studies should explore the motivations for engagement and nonengagement to determine meaningful levels of engagement required for effective intervention.

**Trial Registration:**

ClinicalTrials.gov NCT01092364; https://clinicaltrials.gov/ct2/show/NCT01092364 (Archived by WebCite at http://www.webcitation.org/72V8A4e5X)

## Introduction

### Background

Mobile health (mHealth) technology provides innovative ways to create interventions that may help people lose weight and sustain weight loss [1-3]. mHealth weight loss interventions use mobile user interfaces (eg, short message service text messaging and always-nearby touch screens) to deliver the intervention in the midst of everyday life. They sometimes use the wearable sensing capabilities of the mobile devices as well to gather data and provide tailored feedback to people trying to reduce weight. Some meaningful level of engagement is required to ensure the delivery and receipt of intervention components in research studies.

The effectiveness of behavioral weight loss studies that are delivered in person is known to be moderated by dose [4]. Although not much is known about the relationship between engagement in mHealth interventions and weight outcome, previous studies have shown that a higher engagement is associated with a more favorable study outcome [5,6]. Engagement is a complex concept, and its conceptualization and measurement can vary from study to study. Engagement is sometimes used interchangeably with adherence. Various theoretical models of engagement and adherence have been developed, but not all models have been tested against empirical evidence [7]. In general, these models suggest that individual, environmental, technological, and social support factors may influence user engagement and, subsequently, intervention efficacy.

### Objectives

In this report, we defined engagement specifically as interaction with components of the intervention and then assessed various types of engagement to understand how and whether engagement relates to weight management. Specifically, we defined engagement as the frequency of use of various intervention components of the cell phone app and the attendance of the in-person group sessions and phone counseling calls. We theorized that a higher engagement may reflect individual motivation and lead to a greater commitment for behavioral change and, thus, a higher intervention efficacy in the Cell Phone Intervention For You (CITY) clinical trial.

In the CITY trial, we compared 2 behavioral interventions for weight loss: the cell phone (CP) intervention arm and the personal coaching (PC) arm [8]. Even though neither arm lost a significantly different amount of weight compared with the control arm at 24 months, the PC arm lost the greatest amount of weight at 6 months (*P*<.01). There was also a large variability in weight loss within each arm—some participants lost a substantial amount of weight, whereas others did not. The CITY intervention provides an opportunity to examine engagement because it incorporated various strategies for delivering intervention components, such as using sensor technology for capturing data from a wireless scale, providing tailored feedback, and using prompting as a reminder strategy. In addition, detailed and careful collection of both engagement data and weight data allows for examination of the association between engagement and weight and for examination of factors that may influence engagement. Thus, this work examines (1) the patterns of participant engagement overall, and with key intervention components within each intervention arm, in the CITY trial; (2) the associations of engagement patterns with weight change; and (3) the participant characteristics related to intervention engagement.

## Methods

### Study Design

**Figure 1:**
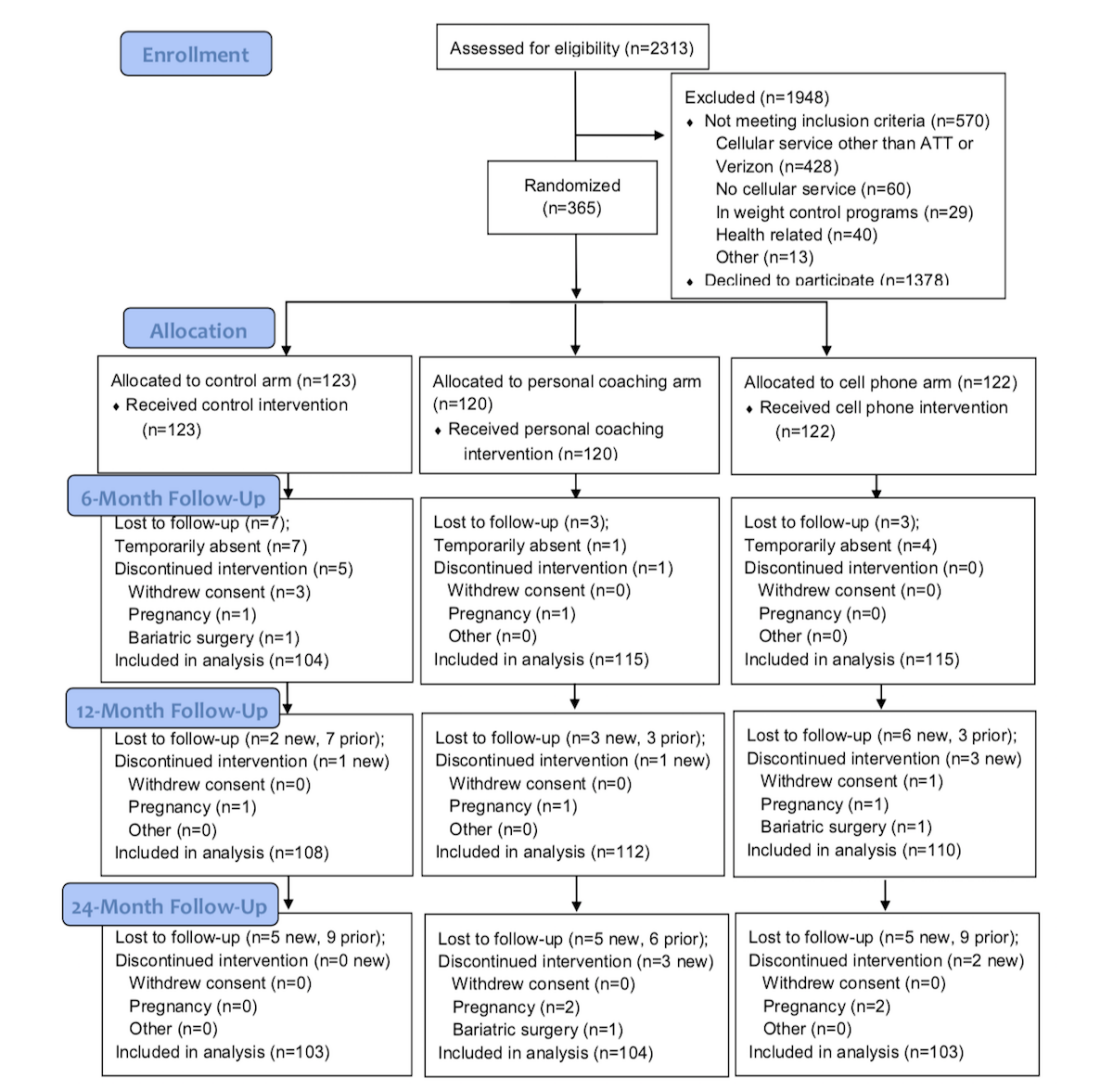
Cell Phone Intervention For You study CONSORT (Consolidated Standards of Reporting Trials) diagram.

The CITY study was 1 of the 7 trials in the Early Adult Reduction of weight through LifestYle Intervention consortium, sponsored by National Heart Lung and Blood Institute (NHLBI, 5U01HL096720) [9]. CITY was approved by the Duke institutional review board and an NHLBI-appointed Protocol Review Committee and Data and Safety Monitoring Board. Enrollment occurred between December 2010 and February 2012. The primary objective of the main CITY study was two-fold: (1) to compare an mHealth intervention delivered by an interactive CP app with usual-care controls and (2) to compare an in-person and phone-supplemented PC intervention enhanced by CP self-monitoring with usual care (Figure 1). For this report, we analyzed data from the 2 intervention arms, not the control arm. The intervention period lasted 24 months, with data collection at baseline and 6, 12, and 24 months post randomization.

### Study Population and Randomization

A total of 365 individuals, aged 18 to 35 years, overweight or obese (body mass index [BMI]>25 kg/m^2^), and currently using a smartphone were enrolled in the study. Individuals were excluded if they were taking weight loss medications, corticosteroids, or had undergone weight loss surgery.

Randomization was stratified by gender and BMI (overweight, BMI≥25-30 kg/m^2^ vs obese, BMI≥30 kg/m^2^) with equal allocation to each of the 3 study arms.

### Interventions

Full descriptions of the CITY study [8] and intervention [10] have been reported elsewhere. The control arm received handouts on healthy eating and exercise for weight management immediately post randomization. No further intervention was offered to the control participants. A summary for each of the 2 active interventions is given below. Both the CP and PC interventions were rooted in theoretical models, and the behavioral framework was based on previous intervention programs that led to significant weight loss in 6 months [11,12].

Behavioral change techniques such as self-monitoring, feedback on behavior, goal setting, problem solving, action planning, behavioral contract, comparison of outcomes, incentive, behavior substitution, habit formation, prompts or cues, modeling of behavior, and shaping knowledge were incorporated into components of the intervention (Multimedia Appendix 1). As self-monitoring has been shown repeatedly to be an important feature of behavioral weight-loss programs [13,14], we emphasized this behavioral strategy in both intervention arms. Participants in the CP arm were prompted by the smartphone app daily to self-weigh, whereas the PC participants were encouraged by their coach to self-weigh during monthly calls. The CP arm also received auditory, vibrotactile, and visual prompting from the app based on a predetermined schedule to record food intake, set goals, and engage in physical activity. The prompting schedule was designed by the intervention team based on collective research experience and the consensus to (1) focus on 1 behavioral technique of self-weighing and (2) emphasize a few key self-monitoring techniques during the first 6 months and then reduce the frequency of prompting over time. We believed that regular prompting initially could help establish new behaviors, but that too much sustained prompting might be disruptive and not sustainable. In addition to the study phone, participants were given a scale (HD-351BT, Tanita Corp, Tokyo, Japan) to record weight readings. The scale wirelessly communicated with the CITY app via Bluetooth and allowed participants to take a measurement without entering data into the smartphone, thereby possibly reducing participant burden [10]. The CITY app uploaded the weight data and other app use data to the research server, and these summary data were available to the study investigators and interventionists. Participants in both of the intervention arms were provided with a smartphone that was used as their personal phone, and they were compensated for monthly smartphone data coverage and for attending the data collection visits. The participants could uninstall the CITY app themselves.

### Personal Coaching Intervention

Participants randomized to the PC intervention arm attended 6 weekly 2-hour group sessions within 2 months of randomization. The sessions included 5 to 10 participants each and were conducted by a coach with registered dietitian training, with multiple years of coaching experience, and trained in motivational interviewing techniques. At the conclusion of all 6 group sessions, participants received a monthly coaching call from the coach for an additional 22 months (21 calls total). No other intervention contact was made between the coach and participants outside of group sessions and monthly phone coaching. To supplement the coaching, PC participants were encouraged to use the CITY smartphone app to track weight, diet, and physical activity. The uploaded weight data allowed the coach to know if a participant was recording weight measurements daily, as recommended, and to discuss such behavior (or lack of it) during the monthly calls.

In contrast to the app used by the CP arm, the app available to PC participants was entirely passive, requiring participants to self-initiate use by opening the app icon. It did not proactively present the user with information, prompt for information, or send reminders to use the app, and thus, it was unlikely to be seen every time participants used their smartphones.

### Cell Phone Intervention

The CP intervention was designed following the same behavioral framework. However, the behavioral framework was implemented for a smartphone, without interaction with a live coach. The delivery mode for the CP intervention was through a fully automated smartphone app that included auditory, vibration, visual, and peripheral prompting intended to encourage use and gather data. This app was designed and developed by the study team. Coaches communicated with the CP participants every 6 months for a quick *check-in* by phone to make sure the smartphone was working properly, but otherwise, the CP intervention was delivered entirely through the smartphone app.

The smartphone app had 24 components within 10 behavioral strategies such as tutorials; tips and news; goal-setting; a buddy system; food tracking; physical activity tracking; feedback and challenge games to increase self-monitoring and physical activity; and an *other* component including components such as sending feedback about the app, sending requests for help to the research team, and updating the app. Multimedia Appendix 1 describes the components that the CP participants were prompted to use and how engagement was tracked from use of those components. The app also included proactive visual, vibratory, and auditory prompting to grab the user’s attention and peripheral display reminders [15] that appear regularly on the lock and home screens and may capture the user’s attention at times. Prompting (both active and peripheral) may be helpful to increase engagement because it (1) might influence general attitude (eg, message sent through prompting: “You have been in the CITY study for 150 days”), (2) remind participants about specific goals (eg, “Take one weight measurement each day”), (3) encourage immediate performance of a task such as self-monitoring (eg, “Track your veggie intake now”) or goal setting (eg, “Set your weekly weight loss goal”), (4) increase knowledge transfer (eg, “Popcorn without butter is a healthy snack”), (5) provide positive reinforcement (eg, “Great job tracking your food”), and (6) deliver social support (eg, “Your weight loss buddy says ‘Good job tracking’”). The prompting was only designed for certain app components for the CP arm. The frequency of prompting for each app component was averaged for each of the 3 study periods (months 1-6, 7-12, and 13-24 months) and is shown in Multimedia Appendix 1.

The visual prompts moved the home screen of the app to the smartphone’s foreground (regardless of what other apps the person might be using at that time), displaying the app content along with a 4 2 seconds audio chime and/or a 4 seconds vibration pattern, depending upon the smartphone’s audio settings at the time. The app also included peripheral always-on reminders, achieved in 2 ways: (1) messages appeared on the smartphone’s lock screen, so that every time the smartphone was used and turned on, a CITY message related to tips or motivation for weight loss was visible and (2) participants were requested to set the CITY app to control their smartphone’s home screen *wallpaper*, so that once the smartphone was unlocked, CITY displayed a message on the smartphone’s app home screen constantly. The wallpaper display included a link to open the main app with a tap. However, the participants could disable the home screen display, and the app could not prevent them from doing so. Participants could also simply cover up the home screen display with other images. As in the PC arm, participants in the CP arm were provided with a wireless scale that could send data to the smartphone app [10].

### Measurement and Recording of Engagement

Programmatically, it is beyond the scope of this report to measure the dosage of most components of the app accurately such as the duration of time the app component was used for but rather only initiation or use of the component. This work, therefore, focuses on the app use as a proxy for the level of engagement. The CITY app logged participant use of every major app component-action (eg, obtaining weight from the scale, dietary tracking, and physical activity tracking) for both intervention arms. There were a total of 18 app component-actions logged for the PC arm and 24 actions logged for the CP arm (see Multimedia Appendix 1) [10]. These data were uploaded daily from the smartphones into the study server. Completion of group sessions and monthly calls in the PC arm were also counted toward engagement and were recorded by the coach into the study database. For this report, we defined engagement as the use of specific app components. For the PC arm only, we additionally defined engagement as attendance at group sessions and phone counseling calls.

### Statistical Analysis

Recognizing that engagement with different components of the intervention demands different amounts of time and effort (ie, attending group session vs weighing self), we evaluated all components of engagement combined and separately for specific app components. For this manuscript, we considered a discrete instance of *use* of each app component to be achieved when a participant completed an important, but also measurable, interaction with that component. In other words, looking at a screen that asks a participant to take a weight reading does not count as use, but entering a weight value when asked to do so does count as use. Engagement data were summarized for each participant for each app component for each day; then, the participant-level data were averaged for the CP and PC arms (separately and combined) over time by month and by 3 specific intervention periods: from baseline to 6 months, 7 to 12 months, and 13 to 24 months. These periods correspond to distinct phases of the intervention with regard to availability and frequency of different app components.

We also examined the relationship between categories of weight change and engagement during the 3 study periods. Weight change was grouped into 4 categories: gained (≥2%), stable (±2%), mild loss (lost ≥2% to <5%), and greater loss (lost ≥5%). These categories were chosen to reflect current guidelines that support 2% to 5% weight loss as clinically meaningful and stable weight as within ±2% of weight gain or loss [16]. One-way analysis of variance (ANOVA) was used to compare each of the 5 selected engagement measures across these weight change categories separately for the CP and PC arms for each intervention period. The 5 engagement measures included (1) mean percentage of days any app component was used (including self-weighing; PC and CP), (2) the mean number of times any app component was used per day (including self-weighing; PC and CP), (3) the mean percentage of days participants self-weighed (PC and CP), (4) the mean completion rate of group sessions (PC only), and (5) the mean completion rate of group sessions and monthly calls combined (PC only). In addition to examining the relationship between categories of weight change and the engagement measures during the 3 intervention periods, we used the Pearson linear correlation coefficient to assess the linear association of these 5 engagement measures with continuous weight change separately for the PC and CP arms for each of the 3 intervention periods.

To understand whether baseline characteristics were associated with higher levels of engagement during the first 6 months of the intervention, we examined the distribution of selected baseline characteristics across quartiles of the mean number of times any app component was used per day (including self-weighing) for the PC and CP arms separately. Baseline characteristics such as age, gender, race, ethnicity, education, income, weight, BMI, energy expenditure, healthy eating index score, and hypertension status were examined. All statistical tests were two-sided, and a *P* value of <.05 was considered statistically significant. All analyses were conducted using SAS 9.4 (SAS Institute, Cary, NC).

## Results

Table 1 describes the baseline characteristics of the participants randomized to the CP and PC arms, combined and separated. On average, participants were 29.3 (SD 4.2) years old, weighed 100.9 (SD 24.3) kg, and had a mean BMI of 35.3 kg/m^2^ (SD 7.9). Approximately 69.8% (169/242) of these participants were female and 37.1% (90/242) were black.

**Figure 2:**
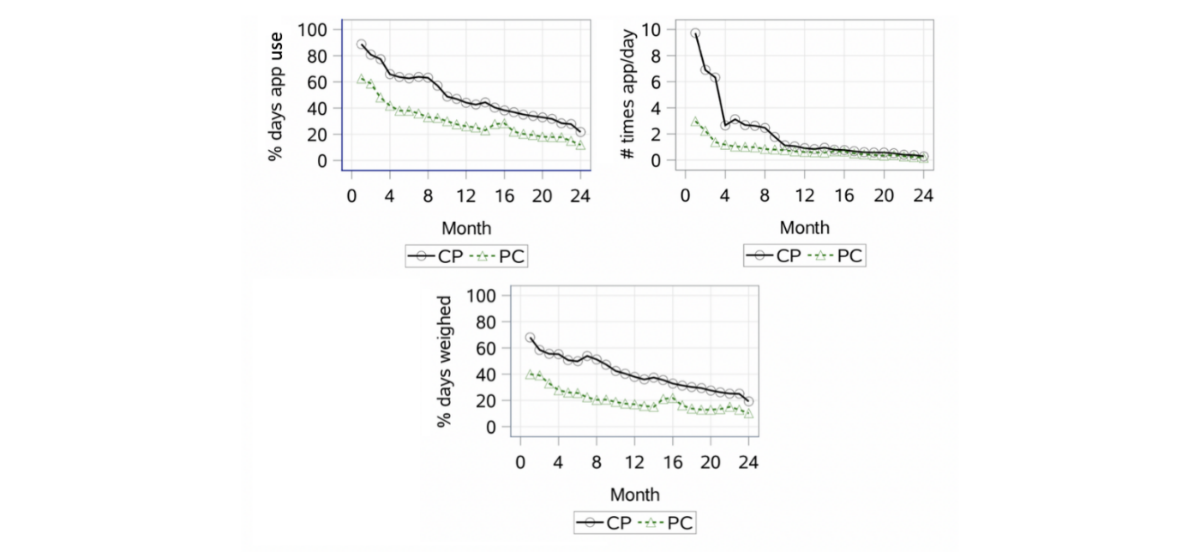
Engagement patterns by arms over time: the pattern of percentage of days any app component, including weighing, was used for each arm over the 24 months, the number of times any app, including weighing, was used, and the percentage of days self-weighing was used. CP: cell phone; PC: personal coaching.

Figure 2 shows the overall pattern of use of the app components by 3 engagement measures: percentage of days apps used including weighing, number of times apps used including weighing, and percentage of days self-weighed in the 2 intervention arms over time because weighing was used the most among all app components. Use was highest during month 1 for both CP and PC arms for all 3 engagement measures. Use dropped substantially during the subsequent 2 to 3 months for the percentage of days used app component and the number of times used app component, and the decrease in use continued until the end of the study. The percentage of days participants self-weighed also decreased steadily after month 1 but not as dramatically as for use of the other components.

Table 2 reports the engagement pattern in the 2 intervention arms during the first 6 months using the 5 engagement measures. The mean percentage of days any app was used (including self-weighing) during the first 6 months was higher (mean 74.2%, SD 20.1) for the CP arm than for the PC arm (mean 48.9%, SD 22.4). The CP arm used any app component (including self-weighing) an average of 5.3 times per day (SD 3.1) during the first 6 months, whereas the PC arm used any app component an average of 1.7 times per day (SD 1.2).

Similarly, during the first 6 months, the CP arm had a higher mean percentage of days self-weighed compared with the PC arm (mean 57.1%, SD 23.7 vs mean 32.9%, SD 23.3). Within the PC arm, engagement in the face-to-face group coaching sessions was high during the first 6 months (mean 93.3%, SD 15.8), as was engagement with monthly calls and group sessions combined (mean 95.2%, SD 9.6).

The pattern of early reduction in engagement is also observed when we examined the engagement pattern for each of the app components by intervention arm and over time. Overall, not counting the use of the CITY home screen component, the CP arm used the app components about 3.24 times a day during the first 6 months, whereas the PC arm used components about 1.08 times a day during the same timeframe (data not shown). Table 3 describes the median daily mean use of the top 10 components by the intervention arm by time. Use for all components was higher in the CP arm than in the PC arm for every period (1-6 months, 7-12 months, and 13-24 months). The most used app component was the main CITY home screen (which could be clicked to launch the rest of the app), implying that the CITY app was actively installed and functioning.

**Table 1:** Demographics by intervention assignment.

Demographic variables		Combined (n=242)	Cell phone (n=122)	Personal coaching (n=120)
**Age (years) at randomization**				
	Mean (SD)	29.3 (4.2)	29.2 (4.2)	29.4 (4.3)
	Median (Q1^a^, Q3^b^)	29.7 (26.3, 32.8)	29.6 (26.6, 32.6)	29.8 (26.2, 33.3)
	Range	19.2-36.0	19.2-36.0	20.0-36.0
Female, n (%)		169 (69.8)	84 (68.8)	85 (70.8)
**Race category, n (%)**				
	White	133 (54.9)	68 (55.7)	65 (54.1)
	Black	90 (37.1)	42 (34.4)	48 (40.0)
	Other	19 (7.8)	12 (9.8)	7 (5.8)
Hispanic ethnicity, n (%)		16 (6.6)	9 (7.3)	7 (5.8)
**Education level, n (%)**				
	Some college or less	90 (37.1)	39 (31.9)	51 (42.5)
	College degree	152 (62.8)	83 (68.0)	69 (57.5)
Working, n (%)		212 (87.6)	107 (88.4)	105 (87.5)
**Weight (kg)**				
	Mean (SD)	100.9 (24.3)	102.4 (25.2)	99.3 (23.4)
	Median (Q1, Q3)	96.1 (83.1, 116.0)	97.8 (83.7, 120.4)	93.5 (83.0, 111.5)
	Range	62.7-189.2	62.7-177.1	64.1-189.2
**Body mass index^c^ (kg/m^**2**^)**				
	Mean (SD)	35.3 (7.9)	35.7 (8.2)	34.9 (7.5)
	Median (Q1, Q3)	33.1 (29.2, 40.8)	33.3 (28.9, 41.6)	32.9 (29.8, 39.3)
	Range	24.9-62.4	25.1-62.4	24.9-58.9

^a^Q1: first quartile.

^b^Q3: third quartile.

^c^Calculated as weight in kilograms divided by height in meters squared.

**Table 2:** Engagement during months 1 to 6 for cell phone and personal coaching intervention arms.

Engagement measures		Cell phone (n=122)	Personal coaching (n=120)
**Percentage of days an app component (including self-weighing) was used**			
	Mean (SD)	74.2 (20.1)	48.9 (22.4)
	Median (Q1^a^, Q3^b^)	78.2 (65.2, 90.1)	46.7 (29.8, 67.4)
	n (range)	114^c^ (16.6-100.0)	110 (7.2-96.7)
**Number of times per day an app component (including self-weighing) was used**			
	Mean (SD)	5.3 (3.1)	1.7 (1.2)
	Median (Q1, Q3)	4.8 (3.2, 6.7)	1.4 (0.9, 2.2)
	n (range)	114 (0.5, 14.7)	110 (0.2, 6.6)
**Percentage of days self-weighed**			
	Mean (SD)	57.1 (23.7)	32.9 (23.3)
	Median (Q1, Q3)	55.2 (38.7, 76.2)	25.1 (13.8, 53.0)
	n (range)	114 (5.0-100.0)	110 (1.7-92.6)
**Percentage of group sessions^d^ completed**			
	Mean (SD)	N/A^e^	93.3 (15.8)
	Median (Q1, Q3)	N/A	100.0 (100.0, 100.0)
	n (range)	N/A	110 (17.0-100.0)
**Percentage of group sessions and monthly calls completed**			
	Mean (SD)	N/A	95.2 (9.6)
	Median (Q1, Q3)	N/A	100.0 (91.5, 100.0)
	n (range)	N/A	110 (46.0-100.0)

^a^Q1: first quartile.

^b^Q3: third quartile.

^c^Participants who dropped out before 6 months were not included in engagement calculations.

^d^Group sessions were only conducted during the first 2 months.

^e^N/A: not applicable. Data were not available for the arm because the component was not offered.

**Table 3:** Median daily mean use of top 10 app components by intervention arm by time.

App component	Example of engagement	Cell phone^a,b^			Personal coaching		
		Months 1-6	Months 7-12	Months 13-24	Months 1-6	Months 7-12	Months 13-24
CITY^c^ app home screen used	Activated CITY home screen	1.06	0.41	0.16	0.76	0.27	0.13
Detailed food tracker used	Entered data in detailed food tracker	0.46	0.14	0.05	0.35	0.04	0.01
Self-weighing component used	Registered weight in app	0.55	0.43	0.22	0.25	0.12	0.08
Sugar-sweetened beverage tracker used	Entered data in SSB^d^ tracker	0.4	0.09	<0.01	0.01	<0.01	<0.01
Physical activity tracker used	Entered data in the PA^e^ tracker	0.31	0.03	<0.01	0.01	<0.01	<0.01
Veggie tracker used	Entered data in veggie tracker	0.22	<0.01	<0.01	0.01	<0.01	<0.01
Meat tracker used	Entered data in meat tracker	0.2	0.01	<0.01	<0.01	<0.01	<0.01
Goals checked off	Checked off a previously set goal	0.1	<0.01	<0.01	<0.01	<0.01	<0.01
Fruit tracker used	Entered data in fruit tracker	0.14	0.06	<0.01	0.01	<0.01	<0.01
“Right now in CITY” viewed	Clicked “Right now in CITY” component	0.04	0.01	<0.01	0.01	<0.01	<0.01

^a^Means were computed as the total number of times each participant used a particular app component during the respective period, divided by the number of days in that period. Then, the median of these means was computed across all participants within cell phone and personal coaching arms separately.

^b^Data were arranged in descending order of use according to the month 1-6 data of the cell phone arm.

^c^CITY: Cell Phone Intervention For You.

^d^SSB: sugar-sweetened beverage.

^e^PA: physical activity.

The second most used app component was the detailed food tracker, with a median daily mean use of 0.46 and 0.35 times per day during the first 6 months in the CP and PC arms, respectively, which is approximately every 2 to 3 days. The detailed food tracker use decreased by more than half during months 7 to 12 and further reduced to about once every 1 to 3 months during the last 12 months in both arms (months 13-24). In the CP arm, where participants were prompted daily to self-weigh, use of the self-weighing component was about every other day during months 1 to 6, which decreased slightly during months 7 to 12 and further reduced to about once every 4 to 5 days during months 13 to 24. For the PC arm participants who did not receive prompting from the smartphone but were encouraged by their coach during monthly calls, the self-weighing component was used about once every 4 days during months 1 to 6 and dropped to about once every 8 days during months 7 to 12 and once every 12 days during months 13 to 24. The CP participants used other components of the app every 2 to 20 days during months 1 to 6, and the use reduced to almost none for the rest of the study. These other components consisted of the sugar-sweetened beverage tracker, physical activity tracker, veggie tracker, meat tracker, goal setter, fruit tracker, and a screen with updates titled *Right Now in CITY*.

Other than the detailed food tracker and self-weighing, the PC participants rarely used any of the rest of the app components that were available to them during the entire study.

**Figure 3:**
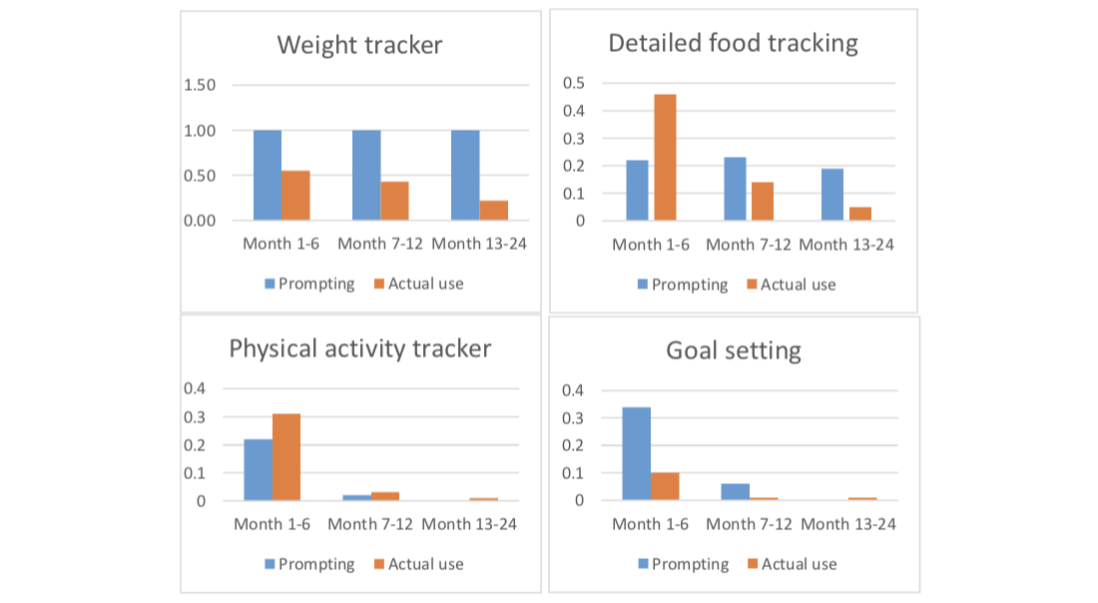
Overall pattern of prompting and actual use of selected app components within the cell phone arm.

Figure 3 illustrates the overall pattern of prompting by selected app components and the actual median daily mean use (Table 3). During the first 6 months, prompting may have helped because all components were consistently used initially. However, use dropped dramatically for all components, possibly related to the reduction in prompting, except for weighing, which was prompted daily until the end of the study. Even though the detailed food tracker was also prompted somewhat regularly through the end of the study, it was prompted less frequently than the weight tracker and use continued to drop after the first 6 months.

Table 4 examines the association between the weight change category by intervention arm across the 3 periods (months 1-6, 7-12, and 13-24) and the 5 measures of engagement: mean percentage of days any app was used (including self-weighing), the mean number of times an app component was used per day (including self-weighing), the mean percentage of days participants self-weighed, the mean completion rate of group sessions for the PC arm, and the mean completion rate of group sessions and monthly calls combined for the PC arm. For each of the 3 periods, weight change was grouped into the same 4 categories mentioned earlier: gained (+2%), stable (±2%), mild loss (lost ≥2% to <5%), and greater loss (lost ≥5%). During the first 6 months, engagement was associated with weight change category when assessed as the mean percentage of days any app component was used, the mean number of times any app component was used per day, or the mean percentage of days self-weighed. These associations persisted until months 7 to 12 for the PC arm. Furthermore, for this arm, the completion of group sessions and group session plus monthly calls combined were not associated with weight change categories. Similarly, when the association between the engagement measures and percentage change in weight change was examined using Pearson correlation coefficients for each period, increased engagement was associated with weight loss (last column in Table 4). The mean percentage of the days any app component was used (CP: *r*=−.213, PC: *r*=−.319), the mean number of times app was used per day (CP: *r*=−.264, PC: *r*=−.308), or the mean percentage of days participants self-weighed (CP: *r*=−.297, PC: *r*=−.354) were each correlated with weight change during the first 6 months.

We also examined selected baseline characteristics of mean number of times the app was used per day during the first 6 months in CP and PC arms separately, across the 4 quartiles. None of the characteristics examined appeared to be related to the varying engagement patterns of the participants (see Multimedia Appendices 2 and 3).

**Table 4:** Five measures of engagement over time (months 1-6, 7-12, and 13-24) by weight change category for cell phone and personal coaching intervention arms.

Engagement measures and time			Weight change categories								*P* value^a^	*r*^b^
			Gained >2%		Gained ≤2% or lost <2%		Lost ≥2% to <5%		Lost ≥5%			
			n	Mean (SD)	n	Mean (SD)	n	Mean (SD)	n	Mean (SD)		
**Percentage of days apps used^c^**												
	**1-6 months**											
		CP^d^	31	74.4 (17.0)	41	68.5 (21.3)	20	75.4 (23.6)	22	83.4 (15.5)	.04	−.213
		PC^e^	16	40.2 (23.0)	39	42.4 (22.5)	29	52.5 (19.6)	26	59.9 (20.4)	.004	−.319
	**7-12 months**											
		CP	24	52.2 (23.6)	46	59.2 (24.3)	24	56.0 (24.9)	11	60.6 (31.5)	.68	.004
		PC	27	24.3 (19.9)	44	31.8 (23.8)	21	44.2 (26.4)	13	27.7 (13.1)	.02	−.124
	**13-24 months**											
		CP	30	28.7 (24.7)	43	40.9 (25.4)	13	34.2 (21.4)	10	43.3 (20.5)	.15	−.127
		PC	43	22.0 (21.2)	32	19.0 (18.8)	15	19.7 (20.7)	8	24.8 (19.6)	.86	.058	
**Number of app uses per day^c^**												
	**1-6 months**											
		CP	31	5.0 (2.5)	41	4.3 (2.5)	20	6.4 (3.8)	22	6.8 (3.5)	.006	−.264
		PC	16	1.2 (1.0)	39	1.3 (0.9)	29	1.9 (1.4)	26	2.3 (1.3)	.001	−.308
	**7-12 months**											
		CP	24	1.7 (1.5)	46	1.8 (1.1)	24	1.7 (1.3)	11	2.2 (1.8)	.70	−.035	
		PC	27	0.5 (0.5)	44	0.8 (0.9)	21	1.3 (1.2)	13	0.6 (0.3)	.02	−.109
	**13-24 months**											
		CP	30	0.6 (0.6)	43	0.7 (0.6)	13	0.6 (0.4)	10	0.8 (0.4)	.56	−.053
		PC	43	0.5 (0.6)	32	0.4 (0.5)	15	0.5 (0.5)	8	0.5 (0.4)	.96	.042
**Percentage of days weighed self**												
	**1-6 months**											
		CP	31	52.1 (22.4)	41	51.9 (23.9)	20	60.8 (25.6)	22	70.2 (18.8)	.01	−.297
		PC	16	24.2 (19.2)	39	26.3 (22.6)	29	35.4 (21.3)	26	45.4 (24.0)	.003	−.354
	**7-12 months**											
		CP	24	40.8 (25.6)	46	50.6 (24.1)	24	48.8 (27.0)	11	52.5 (34.0)	.46	−.031
		PC	27	14.2 (18.6)	44	20.3 (20.2)	21	30.7 (26.1)	13	17.6 (12.5)	.05	−.139
	**13-24 months**											
		CP	30	24.4 (25.2)	43	36.3 (25.8)	13	28.3 (21.8)	10	36.5 (20.6)	.20	−.109	
		PC	43	16.4 (19.8)	32	13.0 (16.2)	15	14.3 (17.8)	8	20.3 (15.9)	.73	.066
**Percentage of group sessions^f^ attended**												
	**1-6 months**											
		PC	16	87.5 (22.3)	39	92.3 (17.8)	29	96.5 (8.2)	26	94.8 (14.1)	.29	−.194
**Percentage of group sessions^f^ and monthly calls attended**												
	**1-6 months**											
		PC	16	90.6 (14.0)	39	94.2 (10.2)	29	97.4 (5.6)	26	96.9 (7.8)	.09	−.246
	**7-12 months**											
		PC	27	98.7 (4.5)	44	90.9 (21.7)	21	95.2 (16.0)	13	100.0 (0.0)	.13	−.066	
	**13-24 months**											
		PC	43	89.6 (18.8)	32	89.8 (18.5)	15	92.9 (15.2)	8	88.6 (18.2)	.92	.019

^a^*P* value for the one-way analysis of variance (ANOVA) test comparing mean engagement measures across weight change categories.

^b^Pearson correlation coefficient for measuring linear association between engagement and percentage change in weight over time (where weight loss is indicated by a percentage change less than zero; a negative correlation indicates a positive association between increased engagement and weight loss).

^c^Includes self-weighing.

^d^CP: cell phone.

^e^PC: personal coaching.

^f^Group sessions only occur in the first 2 months.

## Discussion

### Principal Findings

Our results demonstrate that engagement in mHealth delivery of a behavioral weight loss intervention was associated with weight loss. Our findings suggest that the more the participants used the smartphone app or self-weighed, the more weight loss was observed during the first 6 months of the study for both intervention arms. This association continued to be true for the PC arm into months 7 to 12, but not for the CP arm. Despite the fundamental differences in the time and effort needed in using various components of the smartphone app or in completing personal contacts (ie, group sessions and monthly calls), the finding is consistent—engagement with the intervention is associated with weight loss. It is unclear, however, what level of engagement is required for effective weight loss, and if a different level of engagement is effective for weight loss maintenance.

### Comparisons With Prior Studies

Our findings are consistent with previous research showing that greater engagement with an intervention was associated with greater weight loss, even with different types of engagement measures [17-19]. For example, in a weight loss clinical trial testing the use of interactive voice response (IVR) technology for self-monitoring, 91 participants were randomized to either control or IVR for 12 months [18]. Completion of the IVR calls was significantly correlated with weight loss. Two other studies also found that more daily weighing was associated with greater weight loss during a 6-month intervention [20] or during the first 6 months of an 18-month intervention [21]. In the CITY study, among all components of the smartphone app, a greater percentage of the days participants weighed themselves was independently correlated with greater weight loss in both arms. Other research has also shown that weight loss was associated with greater engagement with participant self-weighing [17,20,22,23]. A 6-month intervention showed that participants in the intervention arm self-weighed more days per week (mean 6.1, SD 1.1) than controls (mean 1.1, SD 1.5), and these participants lost significantly more weight than controls (−6.55% vs −0.35%) [21]. Another 6-month randomized study of 101 participants also showed that weekly self-weighing significantly impacted weight change [24]. These findings demonstrate that mHealth can use behavior change techniques such as self-monitoring effectively for weight loss intervention, with sufficient engagement.

Unlike self-weighing, other components of self-monitoring components such as dietary tracking were only used during the first month and almost never used beyond the first month. This low engagement in dietary tracking may result from low motivation [25,26] or dissatisfaction with the study app; however, we are not able to distinguish the causes. In a study examining differences in dietary intake between participants randomly assigned to monitor their diet via a handheld electronic device or paper journal, no differences were seen between the arms in weight loss, energy intake, or percentage of energy (kcal) from fat [27]. The study showed that adherence to self-monitoring of dietary intake is important for weight loss across several methods of self-monitoring. However, participants using a mobile device recorded twice as many days per week of the self-monitoring diet as those using a paper method [27], and providing feedback was associated with a greater use of self-monitoring. Even though the CITY app included limited feedback within the diet-tracking component, it is unclear how this aspect of the design affected the engagement. Nevertheless, in the aforementioned study, self-monitoring in all 3 groups declined over time, so that by 6 months, only 7 participants (16% of the group) in the smartphone device group continued to record their dietary intake every day (no participants in the diary and Web group had done this) [27]. Thus, long-term adherence is challenging even with early engagement. Tailoring self-monitoring methods that meet users’ needs and circumstances and provide individualized feedback may be helpful in increasing engagement. In addition, future research is needed to develop effective diet tracking strategies that require minimal effort and time, similar to the ease of self-weighing via the Bluetooth scale. Although still in its infancy, technologies including object recognition and voice activation are being actively researched for diet tracking purposes and have the potential to become effective and streamlined strategies.

Indeed, ours and other studies have shown that the engagement dropped drastically, even after the first month, and declined continuously over time [19,28-30]. In a study testing self-monitoring strategies delivered via either IVR or the Web among 180 participants, self-monitoring declined in both modalities over the 24 months of intervention [19]. The decline in long-term engagement may explain, at least partially, why self-weighing was significantly associated with weight change during the first 6 months for both CP and PC arms, and during months 7 to 12 for PC arm only. In an 18-month weight loss study [21], participants were advised to weigh every other day or at least 3 days a week. Adherence to self-weighing only affected weight change during the first 6 months, but not during the remaining 12 months. It is possible that the lack of continued impact of self-weighing on weight change may be due to the decline in self-weighing and/or due to the fact that some participants entered into maintenance mode and, thus, used self-monitoring for a different purpose than during active weight loss phase. As the CITY intervention protocol did not distinguish between initiation and maintenance of weight loss, some participants may weigh less after the first 6 months because they were not actively trying to lose weight but primarily desired maintenance. The switch from an active weight loss mode to a maintenance mode may also explain the substantial drop in percentage of days the app was used in the PC arm from months 0 to 6 to months 7 to 12 among those who lost the most weight (≥5%). This observation was also similar for the self-weighing pattern. This drop in engagement may have contributed to the relapse in weight loss, but this needs to be verified in future studies.

In a 12-month behavioral weight loss study, the self-monitoring pattern of 148 participants also varied and declined over time [30]. Indeed, it may be unrealistic to expect that engagement with any intervention will persist long term. Similar to the 3-stage model used in the medical adherence research (initiation, implementation, and persistence) [31], different strategies may be needed to address engagement needs during different periods of an intervention. Future research should design and test specific strategies to promote and maintain engagement for different stages of intervention, because different types of intervention may require different types of engagement and prompting. Future research should also investigate the effective dose of engagement because there may be a threshold effect. Effective engagement during each app use may be more important than simply more app use [32]. Identifying other factors that contribute to behavior change is important because engagement itself may not be sufficient.

Although traditional personal contact has been perceived as the ideal mode of intervention delivery, in this study, completion of the group sessions alone or combined with monthly calls in the PC arm was not significantly associated with weight change. However, engagement as assessed by overall app use or self-weighing was significantly associated with weight loss, even for the PC arm that received regular and sustained personal support. This finding suggests that mHealth for behavioral interventions could supplement and even enhance interventions based on personal contact, even in the setting of a reduced engagement pattern. Combining mHealth intervention with human support may be more efficient than using either of them alone. This is consistent with a recent study that randomized 102 participants into 3 weight loss intervention arms versus control for 12 weeks: a personal contact arm, an mHealth app-only arm, and a combined arm with personal contact and an mHealth app. The authors reported that the combined personal contact and mHealth app arm was as effective as the personal contact arm and tended to be more effective than the mHealth app arm [33]. Thus, future research should explore novel combination of effective components of conventional and mHealth strategies.

Our data suggest that prompting may be helpful to generate engagement to some degree because the CP arm that received prompting regularly used the smartphone app components more than the PC arm did across all measures of engagement. A meta-analysis of 14 studies with varying designs showed that technology-based prompting had a small to moderate effect on engagement as compared with no-strategy [34]. Participants who received the promptings were significantly more likely to engage with the intervention (relative risk 1.27, 95% CI 1.01-1.60). However, our results suggest that prompting may be helpful only initially, losing impact over time. We speculate that excessive prompting may promote habituation, resulting in reduced use and decreased compliance. The tradeoff is that increased audio, vibration, and visual prompting that interrupts and distracts the user from the current task almost certainly leads to increased user burden and resistance, which would likely reduce acceptability and use of the app and, subsequently, long-term engagement. Habituation may also play a role in interpreting why participants may ignore prompting over time, thus reducing its impact. Future studies are needed to understand how to design smartphone interventions that balance intensity and timing of prompting with stimulation of engagement to maximize utility and minimize burden. Unfortunately, our study was not able to distinguish the difference between the true impact of individual motivation and that of prompting on engagement. Future studies should also examine the impact of different types of prompting on responses and engagement. In this study, we only emphasized regular prompting for weighing; it is possible that consistent prompting of other behavioral change strategies such as physical activity tracking may also yield encouraging engagement patterns that can potentially contribute to effective weight management.

### Strengths and Limitations

This study has several strengths. The engagement data reported here were collected objectively with a smartphone app, and the main outcome of weight was reliably measured at each of the study visits in the clinic and does not rely on self-reporting. Furthermore, this study generated a relatively large amount of engagement data of young adults (n=242) for an extended period (24 months). Another strength of this study is the ability to collect engagement data with each of the cell phone app’s components and with the personal interactions, where the design of the intervention components was based on behavioral theories and prior research evidence.

The study has several limitations that must be considered when interpreting data. Regardless of the reason for joining the CITY study, the varying motivation for weight loss among the participants may have contributed to the varying level of engagement in the intervention arms. Increasing motivation may increase engagement and subsequent weight loss; however, identifying effective level of engagement may also be important for all weight loss studies and programs. Another weakness of the study is that limited study resources prevented development of an intervention app as attractive, polished, and robust as some commercial apps, which could have an impact on engagement. Unfortunately, it was beyond the scope of this study to tease apart whether the reduction in engagement over time may have been due to lack of motivation or challenges with the app design and other technical reasons. Our study compensation for smartphone data coverage, which was offered to both the PC and CP arms, may have incentivized participants to stay in the study but would not contribute to the differences in engagement between the 2 arms. This compensation would not be available to app users if the app were widely deployed. Our compensation, however, did not require any substantial level of engagement or use of app components, and so, participants with low motivation who may have otherwise dropped out of the study may have continued until the end. The overall engagement was lower than expected and desired, but the pattern was consistent with other studies. Messages and tips to encourage healthy lifestyle and weight loss management were delivered through the app home screen, but the smartphone’s operating system prevented the measurement of whether participants covered them up with other app icons or even turned off the messages altogether; this behavior would affect engagement. The fact that engagement dropped substantially early suggests that a more effective intervention that automatically adapts to behavior and self-measured engagement, such as using just-in-time adaptive design, may be needed [35,36]. Another limitation of the study is that participants’ perception of the intervention, which may affect the effectiveness of the intervention, was not included in this assessment. It should also be noted that there is room for optimizing the intervention content that may contribute to a more effective intervention, which may or may not be associated with engagement. For example, optimizing the intervention content may include using a lower carb diet approach instead of a lower fat approach or incorporating a time-restricted eating approach. Future studies should consider not only effective behavioral strategies but also combining those with additional evidence-based dietary and lifestyle approaches for weight loss.

### Conclusions

In this study, engagement assessed using different measures was associated with weight loss. Nevertheless, engagement declined over time at varying rates for different intervention engagement components. This study suggests that a variety of strategies may be needed during different stages of an intervention to increase and sustain engagement required for intervention effectiveness. Self-weighing was associated with weight loss regardless of the baseline characteristics of the participants, suggesting that an effective weight loss program may not need to include multiple behavioral strategies. Focusing on a single effective strategy in conjunction with prompting may be better than offering more components that most participants may not use. Future studies should clarify the definition of effective engagement. In addition, future studies should explore the motivations for participant engagement and nonengagement to design effective strategies for addressing those specific challenges.

## Appendix

Multimedia Appendix 1 Description of the major components of the Cell Phone Intervention For You app, the user actions within each component that count toward engagement, and the prompting frequency for selected components.

Multimedia Appendix 2 Baseline characteristics of cell phone participants by quartile of mean number of apps used per day (including self-weighing) in the first 6 months.

Multimedia Appendix 3 Baseline characteristics of personal coaching participants by quartile of mean number of app uses per day (including self-weighing) in the first 6 months.

Multimedia Appendix 4 CONSORT‐EHEALTH checklist (V 1.6.1).

## Abbreviations

ANOVA: analysis of variance
BMI: body mass index
CITY: Cell Phone Intervention For You
CP: cell phone
IVR: interactive voice response
mHealth: mobile health
NHLBI: National Heart Lung and Blood Institute
PC: personal coaching
