# Supplementary material for: The Association Between Engagement and Weight Loss Through Personal Coaching and Cell Phone Interventions in Young Adults: Randomized Controlled Trial
Source: JMIR Mhealth Uhealth. 2018 Oct 18;6(10):e10471. doi: 10.2196/10471 (PMC6245957; doi:10.2196/10471)
Supplement: Multimedia Appendix 1 [file mhealth_v6i10e10471_app1.pdf]

Multimedia Appendix 1. Description of the major components of the Cell Phone Intervention For You app, the user actions within each component that count toward engagement, and the prompting frequency for selected components.

App component (underlying behavior change technique)[a]	Brief description of the component	Actions counted as engagement[a]	Frequency of prompting in the cell phone arm, times/day[b]		
			Month 1-6	Month 7-12	Month 13-24
Tutorial/ guidelines (skill building)	Shows nine 2-8 min orientation and coaching videos about weight loss strategies; lists lifestyle guidelines	*Viewed tutorial by clicking "Tutorial"; clicked on "Guidelines" component*	0.0	0.0	0.0
Phone 'live wallpaper' displaying tips, links, and facts (health consequences; instruction on how to perform a behavior, prompts and cues)	Presents short messages, tips for healthy living, relevant news, jokes, and motivational quotes on the phone's lock screen and home screens; active constantly and visible when phone is being unlocked	Ran the CITY app with it set to be the home screen/ live wallpaper	0.0	0.0	0.0

Weighing (self-monitoring of behavior; feedback on behavior)	Records weight; presents a brief tailored feedback, a historical weight graph, and a weight projection	*Registered weight in app*	1 (daily)	1 (daily)	1 (daily)
Food tracking (self-monitoring of behavior; feedback on behavior)	Records food intake for a whole day and provides balance of calories toward goal	*Entered data in "Detailed food tracker"*	0.22 (every 5 days)	0.23 (every 5 days)	0.19 (every 5 days)
	Tracks servings of sugar-sweetened beverage intake for the day	*Entered data in "Sugar-sweetened beverage (SSB) tracker"*	0.18 (every 5 days)	0.04 (every 25 days)	0.0
	Tracks servings of veggie intake for the day	*Entered data in "Veggie tracker"*	0.08 (every 12 days)	0.0	0.0
	Tracks servings of meat intake for the day	*Entered data in "Meat tracker"*	0.1 (every 10 days)	0.0	0.0
	Tracks servings of fruit intake for the day	*Entered data in "Fruit tracker"*	0.08 (every 12 days)	0.04 (every 25 days)	0.0
	Tracks the healthfulness and portion of the meals and snacks for the day; extremely simple	*Entered data in "Healthy meal tracker"*	NA[c]	0.07 (every 14 days)	0.19 (every 5 days)

Physical activity tracking (self-monitoring of behavior; feedback on behavior)	Tracks physical activity type and minutes of each activity	*Entered data in "Physical activity tracker"*	0.22 (every 5 days)	0.02 (every 48 days)	0.0
Buddy system (social support, behavioral contract)	Pairs each participant randomly with a "buddy" each month; permits buddies to send and receive messages to encourage, advise, and offer tips without revealing identity	Viewed "Buddy profile"; viewed own profile	NA	NA	0.0
Goal setting (goal setting; review behavioral goal; action planning; problem solving)	Encourages participants to set goals for any timeframe of their choice; tracks completion of each goal	*Checked off a previously set goal; set a new goal*	0.34 (every 3 days)	0.06 (every 16 days)	0.0
	Encourages exploration of barriers to achieving goals	*Opened "Barrier" component*	0.08 (every 12 days)	0.04 (every 25 days)	0.0

Countdown (goal setting; review behavior goal)	Starting 4.5 months from the end of the study, counts down days left in study, and allows setting a weight goal; continually displays goal in the phone’s notification bar	Opened “Countdown” component; set a goal weight in “Countdown”	NA	NA	0.0
Challenge/ game (comparison of behavior; behavioral substitution)	Encourages participants to move with a “Move it challenge” that intensifies each day	Responded to a “Move it” challenge	NA	NA	0.54 (every other day)
Rewards and feedback (incentive, nonspecific reward)	Sends rewards via the phone’s notification bar after participants accomplish certain tracking; shows installments of a serial of cartoons, links to weight loss tips, and entertaining blog entries	None counted	0.0	0.0	0.0

Other		*Clicked "Right now in CITY" component; clicked "News" component; clicked "Update" component; sent a request for help; sent in feedback about the app*	0.0	0.0	0.0

[a]The italicized components were available to the personal coaching arm but no prompting was delivered.

[b]The prompting frequency was averaged for each of the app components during each of the 3 study periods. Prompting for 2 components within the goal setting was jointly delivered. A value of 0 indicates that the component was available but there was no prompting. A value of 1 indicates that the participants were prompted to use the component once a day. A value less than 1 indicates that the component was prompted less than once a day, for example, a value of 0.2 indicates the prompting was approximately once every 5 days. Approximate interpretation of the prompting frequency is given in parentheses.

[c]NA indicates that the component was not available to the participants.
