# Supplementary material for: The Association Between Engagement and Weight Loss Through Personal Coaching and Cell Phone Interventions in Young Adults: Randomized Controlled Trial
Source: JMIR Mhealth Uhealth. 2018 Oct 18;6(10):e10471. doi: 10.2196/10471 (PMC6245957; doi:10.2196/10471)
Supplement: Multimedia Appendix 2 [file mhealth_v6i10e10471_app2.pdf]

Multimedia Appendix 2. Baseline characteristics of Cell Phone participants by quartile of mean number of apps used per day (including self-weighing)[a] in first six months

	First Quartile (≤3.2)	Second Quartile (>3.2 & ≤4.8)	Third Quartile (>4.8 & ≤6.7)	Fourth Quartile (>6.7)
Total, no.	29	28	29	28
Age at Randomization (years)				
mean (SD)	28.8 (4.3)	29.9 (4.4)	29.0 (4.2)	29.1 (3.8)
median (Q1, Q3)	28.6 (26.5, 31.4)	31.5 (27.5, 33.2)	29.6 (26.2, 32.4)	28.9 (26.9, 31.7)
n (min, max)	29 (19.2, 36.0)	28 (19.3, 35.2)	29 (19.8, 35.3)	28 (20.4, 35.7)
Female, no. (%)	12 (41.4)	22 (78.6)	24 (82.8)	21 (75.0)
Race Category, no. (%)				
White	16 (55.2)	11 (39.3)	15 (51.7)	20 (71.4)
Black	10 (34.5)	16 (57.1)	10 (34.5)	5 (17.9)
Other	3 (10.3)	1 (3.6)	4 (13.8)	3 (10.7)
Race, no. (%)				
White	16 (55.2)	11 (39.3)	15 (51.7)	20 (71.4)
Non-white	13 (44.8)	17 (60.7)	14 (48.3)	8 (28.6)
Hispanic Ethnicity, no. (%)	2 (6.9)	0 (0.0)	2 (6.9)	4 (14.3)
Education Level, no. (%)				
Some college or less	11 (37.9)	10 (35.7)	7 (24.1)	9 (32.1)
College Degree	18 (62.1)	18 (64.3)	22 (75.9)	19 (67.9)
Income Category, no. (%)				
<24,999	6 (21.4)	10 (35.7)	8 (28.6)	11 (40.7)
25,000 - 49,999	14 (50.0)	12 (42.9)	15 (53.6)	10 (37.0)

Multimedia Appendix 2. Baseline characteristics of Cell Phone participants by quartile of mean number of apps used per day (including self-weighing)[a] in first six months

	First Quartile (≤3.2)	Second Quartile (>3.2 & ≤4.8)	Third Quartile (>4.8 & ≤6.7)	Fourth Quartile (>6.7)
50,000 - >100,000	8 (28.6)	6 (21.4)	5 (17.9)	6 (22.2)
In a Committed Relationship, no. (%)	20 (69.0)	15 (53.6)	19 (65.5)	13 (46.4)
Weight in kg				
mean (SD)	102.1 (23.4)	107.5 (29.5)	95.1 (20.8)	100.5 (20.2)
median (Q1, Q3)	101.8 (78.6, 118.4)	98.1 (83.0, 129.0)	93.5 (80.1, 104.1)	99.3 (85.4, 115.6)
n (min, max)	29 (67.9, 150.6)	28 (70.4, 163.0)	29 (62.7, 137.8)	28 (66.0, 141.3)
BMI (kg/m^2)				
mean (SD)	34.7 (7.1)	38.3 (10.4)	34.0 (7.8)	35.3 (7.0)
median (Q1, Q3)	32.6 (29.2, 39.7)	34.9 (28.9, 49.5)	32.2 (28.9, 37.4)	34.8 (29.0, 41.0)
n (min, max)	29 (26.2, 50.9)	28 (25.1, 62.4)	29 (25.2, 52.8)	28 (25.8, 47.8)
Weekly energy expenditure in leisure time phys. activity that includes stairs climbed				
mean (SD)	1,099.5 (1,212.1)	1,030.7 (1,736.7)	980.2 (1,268.3)	1,109.5 (1,397.5)
median (Q1, Q3)	612.0 (344.0, 1,394.5)	188.0 (84.0, 1,016.0)	576.4 (112.0, 979.3)	659.5 (192.0, 1,548.0)

Multimedia Appendix 2. Baseline characteristics of Cell Phone participants by quartile of mean number of apps used per day (including self-weighing)[a] in first six months

	First Quartile (≤3.2)	Second Quartile (>3.2 & ≤4.8)	Third Quartile (>4.8 & ≤6.7)	Fourth Quartile (>6.7)
n (min, max)	25 (0.0, 4,671.0)	21 (28.0, 6,103.0)	28 (0.0, 5,224.0)	27 (56.0, 5,928.0)
HEI Score				
mean (SD)	51.0 (3.6)	52.5 (4.0)	53.0 (5.2)	50.5 (3.9)
median (Q1, Q3)	50.7 (48.6, 53.3)	51.7 (49.1, 54.9)	53.6 (48.0, 56.7)	51.1 (47.1, 52.9)
n (min, max)	26 (44.2, 59.8)	25 (45.4, 60.5)	26 (45.2, 62.0)	28 (42.3, 57.2)
Hypertension[b], no. (%)	5 (17.2)	6 (21.4)	5 (17.2)	3 (10.7)

Abbreviations: CELL PHONE, Cell Phone Intervention; PC, Personal Coaching Intervention; SD, standard deviation; Q1, 1st quartile; Q3, 3rd quartile; BMI, body mass index; HEI, healthy eating index.

[a]This measure was calculated by taking the average (in the first six months) of a binary indicator for each participant of whether they used at least one app or not, then averaging this across all participants.

[b]Resting SBP > 140 or resting DBP > 90 or taking BP medication.
